# Virtual reality tumor navigated robotic radical prostatectomy by using three‐dimensional reconstructed multiparametric prostate MRI and ^68^Ga‐PSMA PET/CT images: A useful tool to guide the robotic surgery?*

**DOI:** 10.1002/bco2.16

**Published:** 2020-05-09

**Authors:** Abdullah Erdem Canda, Sertac Fatih Aksoy, Emre Altinmakas, Ersin Koseoglu, Okan Falay, Yakup Kordan, Barbaros Çil, Mevlana Derya Balbay, Tarik Esen

**Affiliations:** ^1^ Department of Urology School of Medicine Koç University Istanbul Turkey; ^2^ Collaboration Space Sabancı University Istanbul Turkey; ^3^ Department of Radiology School of Medicine Koç University Istanbul Turkey; ^4^ Department of Nuclear Medicine School of Medicine Koç University Istanbul Turkey; ^5^ Department of Urology VKF American Hospital Istanbul Turkey

**Keywords:** 3D reconstruction, augmented reality, radical prostatectomy, robotic, training, virtual reality

## Abstract

**Objectives:**

To evaluate the use and benefits of tumor navigation during performing robotic assisted radical prostatectomy (RARP).

**Patients and Methods:**

Borders of the visible tumor(s) was/were and surrounding structures marked on multiparametric prostate magnetic resonance imaging (mpMRI) and ^68^Ga‐labeled prostate‐specific membrane antigen ligand using positron emission computed tomography (Ga68 PSMA‐PET/CT). Three dimensional (3D) reconstruction of the images were done that were transferred to virtual reality (VR) headsets and Da Vinci surgical robot via TilePro. Images were used as a guide during RARP procedures in five cases. Indocyanine green (ICG) guided pelvic lymph node dissection (n = 2) and Martini Klinik Neurosafe technique (n = 2) were also applied.

**Results:**

Mean patient age was 60.6 ± 3.7 years (range, 56‐66). All VR models were finalized with the agreement of radiologist, urologist, nuclear physician, and engineer. Surgeon examined images before the surgery. All VR models were found very useful particularly in pT3 diseases. Pathological stages included pT2N0 (n = 1), pT3aN0 (n = 1), pT3aN1 (n = 2), and pT3bN1 (n = 1). Positive surgical margins (SMs) occurred in two patients with extensive disease (pT3aN1 and pT3bN1) and tumor occupied 30% and 50% of the prostate volumes. Mean estimated blood loss was 150 ± 86.6 cc (range, 100‐300). Mean follow‐up was 3.4 ± 1.7 months (range, 2‐6). No complication occurred during perioperative (0‐30 days) and postoperative (30‐90 days) periods in any patient.

**Conclusions:**

3D reconstructed VR models by using mpMRI and Ga68 PSMA‐PET/CT images can be accurately prepared and effectively applied during RARP that might be a useful tool for tumor navigation. Images show prostate tumors and anatomy and might be a guide for the console surgeon. This is promising new technology that needs further study and validation.

## 1 INTRODUCTION

Prostate cancer (PCa) is the most common malignant disease in men leading to cancer‐related deaths in the United States.^1^ With rapid dissemination of robotic platform worldwide, robotic assisted radical prostatectomy (RARP) has become the most commonly applied surgical method in PCa surgery.^2^, ^3^ Multiparametric prostate magnetic resonance imaging (mpMRI) provides the surgeons with important information about the prostate anatomy, location of the tumor(s), presence of extraprostatic extension (EPE) and involvement of seminal vesicles and neurovascular bundles (NVBs). ^68^Ga‐labeled prostate‐specific membrane antigen ligand using positron emission computed tomography (Ga68 PSMA‐PET/CT) is also used in the evaluation of the patients with PCa in terms of location of the cancer in the prostate, lymph node (LN) involvement and systemic metastasis. Augmented reality (AR) and 3‐dimensional (3D) imaging technology were used in robotic surgery for tumor navigation and precision surgery.^4^ Herein, we present our experience and results in five patients using 3D reconstructed prostate images with index lesion/tumor marked with different colours using mpMRI and Ga68 PSMA‐PET/CT images transferred to Da Vinci surgical console (Intuitive Surgical Inc., Sunnyvale, CA, USA) via TilePro and also to virtual reality (VR) headsets used for tumor navigated RARP.

## 2 PATIENTS AND METHODS

Overall, five patients with the diagnosis of prostatic adenocarcinoma underwent RARP and bilateral pelvic lymph node dissection (BPLND) were included in this study (Table 1). All five patients were operated by the same surgeon (AEC) with previously published technique.^5^ Median patient age was 60 years (range, 56‐66). All patients had mpMRI and Ga68 PSMA‐PET/CT imaging before the RARP procedures.

**Table 1:** Preoperative patient characteristics, preoperative findings, and robotic assisted radical prostatectomy outcomes

Pt Nr	Age (years)	Serum PSA (ng/mL), DRE	Nr of (+) cores with PCa, Localization, GG	mpMRI, PIRADS (P) lesion, Tumor localization	Ga68 PSMA‐PET/CT	Surgeon's comments on the use of 3D VR navigated RARP surgery	Dominant tumor location, Volume, GG	EPE	pT stage, SM, LN status
**1**	60	10	5/10 with PCa	2.9 cm P5 lesion, (anterior transitional zone, apex‐midgland)	The most intense uptake seen in right posterior of mid‐gland also extending into the rectum, including the capsule	Extremely useful and helpful in achieving negative SMs particularly at apex VR images were extensively used Surgeon followed tumor location and borders by checking the VR images Surgeon estimated possible location of the anterior transition zone extensive tumor that guided tissue dissection Images were helpful to decide performing right non‐NVB sparing and left complete‐NVB	Anterior, right posterolateral, apex to base	Extensive: Right posterolateral and right bladder neck	pT3a, ECE (+): extensive right posterolateral and bladder neck, SM (−), LN (+): (2/25) max.1.2 cm left
		Benign prostate	Site not known	0.8 cm P4 lesion (left posterior midgland)			12 cm^3^		
			GG2				GG3		
**2**	58	7	6/12 with PCa	2.2 cm P5 lesion (midgland‐basis) suspicious for a T3 tumor	Bilateral PSMA uptakes, bilateral medial side of basis, right lateral side and left posterior side of mid‐gland, most intense in left posterior of mid‐gland including the capsule	Extremely useful and helpful in achieving negative SMs Console surgeon extensively used VR images to follow the tumor location and tumor borders while performing dissection particularly on the left posterior side Images showed that tumor occupied a significant space in the left peripheral prostatic lobe suggesting EPE Images were useful to make the decision of performing complete bilateral NVB sparing with careful dissection on the left	Left posterolateral apex and midgland	Left posterolateral, multiple areas	pT3a, ECE (+): left posterolateral, SM (−), LN (0/15)
		Irregular and hard left side (cT3) and benign right side of prostate	All on the left side				6.2 cm^3^		
			GG3				GG3		
**3**	66	6.2	10 cores with PCa	4 cm P5 lesion (entire left lobe extending to the left SV and left NVB)	Bilateral PSMA uptakes in the prostate, more intense in medial side of basis, left lateral side of mid‐gland and left medial side of apex	Console surgeon estimated that tumor occupied major part of the left peripheral prostatic lobe before and during surgery by following the VR images Visible extraprostatic extension reaching to levator muscle on the left side was shown on the 3D reconstructed images Images were useful to make the decision of performing non‐NVB sparing surgery on the left side A complete NVB sparing was performed on the right side	Occupying majority of the left lobe (from apex to base) and extending to right lobe	Bilateral extensive from apex to base	pT3a, ECE (+): Bilateral extensive from apex to base, SM (+): 2 cm, LN (+): (1/15) left 0.4 cm
		Left lobe stone hard with mobile rectal mucosa (cT3), right lobe hard/irregular (cT2)	5/5 on the left side		Multiple millimeter sized Ga68 uptakes were identified on left iliac LN area at the level of the left ureter crossing iliac artery that might suggest metastasis		50% of the prostate volume		
			3/5 on the right side				GG3		
			GG2						
**4**	56	11.5	15 cores with PCa	0.5 cm P4 lesion (left posterior midgland)	PSMA uptakes, left lateral‐medial sides of mid‐gland, left medial side of apex	Surgeon felt the use of VR reconstructed images were not as useful as the other patients due to probably having low tumor volume and pT2 disease as this was a rather easy RARP case to perform compared to the others	Left posterolateral midgland	–	pT2, ECE (−), SM (−), LN (0/9)
		Benign prostate	(12/12 cores systematic and 3/4 cores lesion)				1.5 cm^3^		
			GG1				GG1 GG1		
**5**	64	27	4/10 cores with PCa	3.5 cm P5 lesion (left posterior midgland‐basis. Suspicion for left SV and NVB involvement)	PSMA uptakes in left lateral‐medial sides of mid‐gland and left medial side of apex	Console surgeon extensively used VR images to follow the tumor location and tumor borders extending from left apex to base while performing dissection on the left posterior side Console surgeon knew that tumor occupied a major part of the left peripheral prostatic lobe before and during surgery by following the VR images that helped making the decision on left non‐NVB sparing and right complete‐NVB sparing surgery	Left apex to base on the left posterolateral prostate	Extensive from left apex to base	pT3b, ECE (+): Extensive from left apex to base, SM (+): 2 mm, LN (+): (5/19) left max 2 cm
		Stone hard left side of the prostate (cT3) and benign right side	All on the left side				30% of the prostate volume		
			GG3				GG2		

Abbreviations: 3D, three dimensional; DRE, digital rectal examination; EPE, extraprostatic extension; ePLND, extended pelvic lymph node dissection; Ga68 PSMA‐PET/CT, ^68^Ga‐labeled prostate‐specific membrane antigen ligand using positron emission computed tomography; GG, Gleason grade; LN, lymph node; mpMRI, multiparametric prostate magnetic resonance imaging; PSA, prostate specific antigen; Pt Nr, patient number; pTN, pathologic T and N stage, RARP, robotic assisted radical prostatectomy; SM, surgical margin; VR, virtual reality.

Martini Klinik Neurosafe RARP surgical technique^6^ was applied in two patients (patient number 2 and 4). Indocyanine green (ICG)‐fluorescence guided BPLND was performed in two patients (patient number 3 and 5) where, following abdominal port placement, side‐docking was made by using Da Vinci‐xi surgical robot and our interventional radiologist performed transrectal ultrasound (TRUS) and injected 1 cc ICG into each prostate lobe via transperineal route (5 mg ICG diluted with 2 mL distilled water).^7^, ^8^

### 2.1 mpMRI technique

mpMRI studies were performed on a 3.0 Tesla MRI machine (3T Magnetom Skyra, Siemens AG Healthcare, Erlangen, Germany). In addition to conventional mpMRI sequences suggested by PIRADS version v2.1, a 3‐dimensional T2‐weighted space sequence on axial plane was also obtained in order to use for 3D modeling. The technical parameters were as follows; slice thickness (ST): 1 mm, echo time (TE): 101, repetition time (TR): 1500, and flip angle (FA): 135. Postprocessings were performed on this particular sequence on a PACS system (GE PACS Healthcare System). Borders of the peripheral and transitional zones were drawn on each slice by using free‐hand region of interest (ROI). Similarly, borders of the lesions which were pathologically known to be adenocarcinoma were also drawn on 3D‐T2W images. To avoid possible over‐ and underestimation, extension of the tumors were evaluated on other mpMRI sequences as well.

**Figure 1:**
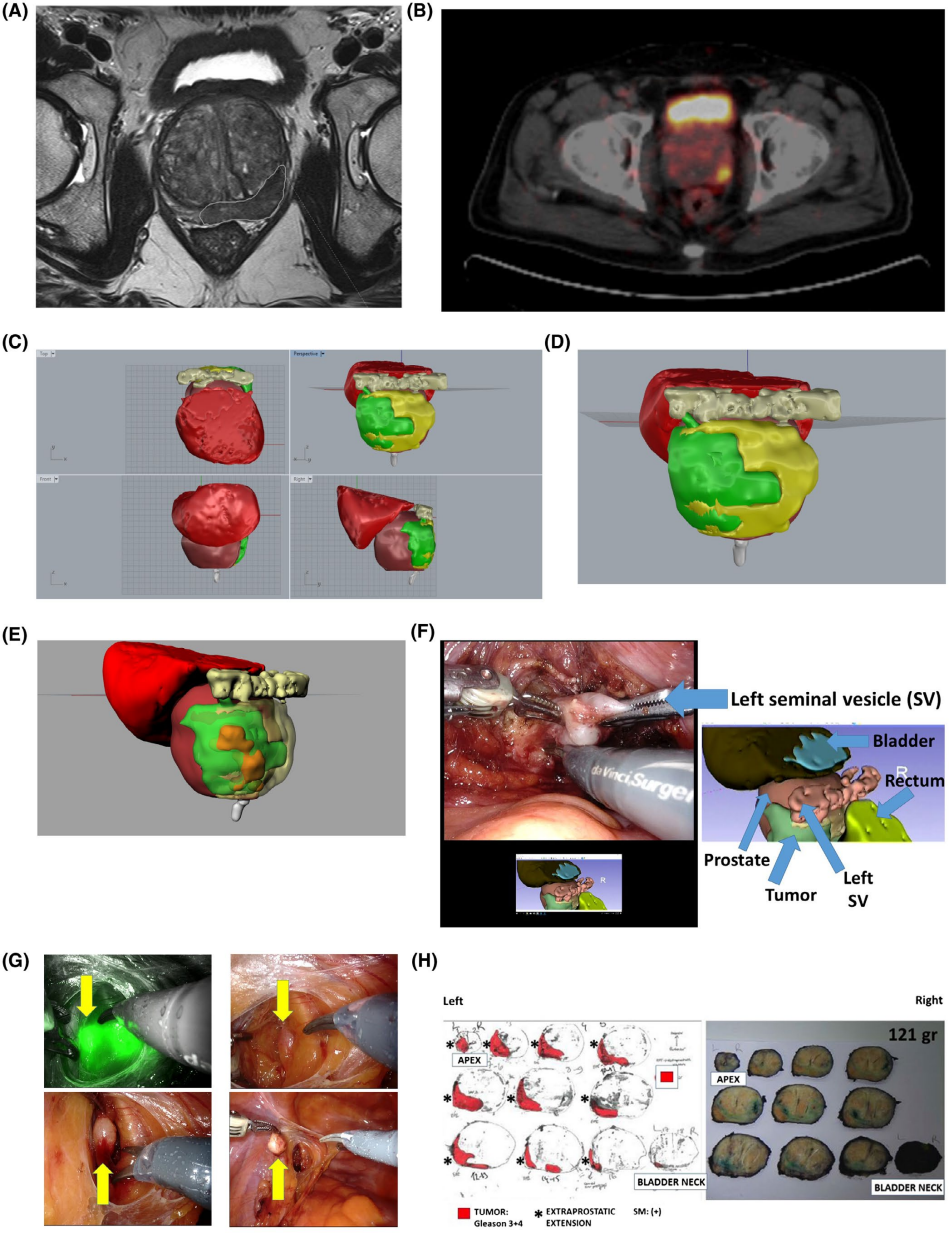
A, mpMRI image. Axial T2‐weighted space sequence shows a 3.5 cm PI‐RADS 5 lesion in the left posterior peripheral zone at the level of midgland‐basis. There is suspicion of left seminal vesicle and NVB involvement. Borders of the lesion which was pathologically known to be adenocarcinoma was drawn. B, Ga68 PSMA‐PET/CT: PSMA uptakes were seen in left lateral‐medial sides of mid‐gland and left medial side of apex with 3.21 SUVmax. C, 3D reconstructed image of the prostate. Left up (*yx* axis): appearance from the top, left down (*zx* axis): appearance from front, right down (*zy* axis): appearance from right, right up (*xyz* axis, perspective). Green: tumor on MRI, yellow: peripheral zone, purple: anterior‐transition zone, red: bladder. D, 3D reconstructed image of the prostate (maximized perspective, *xyz* axis). Green: tumor on MRI, yellow: peripheral zone, purple: anterior‐transition zone, red: bladder. Tumor involving almost the entire left half of the prostate gland with obvious extraprostatic extension is visible (green). E, 3D reconstructed image of the prostate with tumor on Ga68 PSMA‐PET/CT overlap in addition to mpMRI (maximized perspective, *xyz* axis). Orange: 68Ga‐PSMA uptake area, Green: tumor on MRI, yellow: peripheral zone, purple: anterior‐transition zone, red: bladder. F, Real time use of 3D reconstructed image of the prostate during RARP and intraoperative surgical appearance. Due to the possible involvement of left seminal vesicle and NVB by the tumor that also appears in the 3D images, console surgeon did not preserve left NVB and did a careful dissection at the level of left seminal vesicle. G, ICG guided pelvic LN dissection (left side). Please note ICG(+) LN that was excised and sent for intraoperative pathological frozen evaluation that was reported as metastatic. H, Postoperative pathology mapping of the prostate

### 2.2 Ga68 PSMA‐PET/CT technique

The vertex to middle thigh body scan was done with Discovery IQ PET/CT (GE Healthcare, USA) (3 min per bed for PET, 50‐200 mAs, 120 kV for CT) 50 min. after 68Ga‐PSMA‐11 injection dose of 0.05 mCi/kg and following the body image, pelvic region prone image was taken after emptying the bladder. Images were reconstructed with Q. Clear uses the Block Sequential Regularized Expectation Maximization algorithm.

The images were interpreted in AW work station of GE by a nuclear medicine physician. Any focal 68Ga‐PSMA uptake higher than surrounding activity not associated with a known site of physiological uptake and with a corresponding morphological abnormality on CT was considered pathological and suspicious for malignancy. All pathological uptakes were analyzed with regard to their location and their maximum standardized uptake value (SUVmax).

### 2.3 3D image reconstruction of the prostate

Axial T2W 3D‐TSE sequence was used in mpMRI. Borders of the tumor(s) were marked by our uro‐radiologist. Ga68 PSMA‐PET/CT images were evaluated by nuclear medicine physician. 3D images of the prostate were created by engineer. In order to obtain the accurate segmentation of the tumor(s) and surrounding anatomical structures, interactive medical image segmentation methods were used such as, growcut algorithm and morphological contour interpolation. Simple region growing techniques were used after primary applications for improving the results of the initial methods. Urinary bladder, prostate (peripheral zone was separated), and urethra were segmented to give a better understanding.^9^ Tumor(s) on mpMRI and Ga68 PSMA‐PET/CT images were marked with different colors.

Created images were successfully transferred to VR headsets and/or to Da Vinci surgical system via TilePro to be used as a guide for the operating console surgeon. During the RARP procedure, our engineer or the surgeon himself used mouse to rotate the image manually in order to present the area of interest to the console.

Following completion of each RARP procedure, surgical comments were recorded for each patient regarding the use of the VR images during surgery.

## 3 RESULTS

Detailed characteristics of the patients such as age, prostate‐specific antigen (PSA), digital rectal examination (DRE) findings, mpMRI and Ga68 PSMA‐PET/CT images, biopsy findings together with surgeon's impressions preoperatively and histopathological results of RARP procedures are given in Table 1. All but one patients (patient 4) had TRUS guided biopsies without pre biopsy mpMRI. Remaining four patients had their mpMRI imaging 6 weeks following the biopsy. All patients received RARP with extended BPLND by a single surgeon (AEC). Median estimated blood loss was 100 cc (range, 100‐300). Postoperative follow‐up was uneventful for all patients and were all discharged on postoperative day‐3 or 4. No complication occurred during perioperative (0‐30 days) and postoperative (30‐90 days) periods in any patient.

Two patients had bilateral complete while 3 others had only unilateral NVB preservation. Three patients (patients 1, 2, and 4) had also nondominant tumor foci with ≤Gleason grade 2 (GG) tumors. Neurosafe was applied in two patients (patient number 2 and 4) with negative surgical margins (SM). ICG was given to patients 3 and 5, where LNs were reported as benign (false negative) in patient 3 and as metastatic in patient 5. Following RARP, tumors in patients 1 and 3 were upgraded; 2 and 4 did not change while it was downgraded in patient 5. Only patient 4 had organ confined disease and patients 1, 3, and 5 already had developed LN metastases. Despite extensive extracapsular extension (ECE) in four patients (patients 1, 2, 3, and 5) SMs were tumor free in two (patients 1 and 2) and only a 2 mm positive in one (patient 5). mpMRI and PSA correctly diagnosed ECE in three patients (patients 1, 2, and 5) of four patients and in two (patients 1 and 2) of four patients, respectively. Only mpMRI correctly diagnosed the patient 5 with positive seminal vesicle invasion (SVI) but was false positive in another one (patient 3). Only one (patient 3) out of three LN positive patients was predicted by Ga68 PSMA‐PET/CT. mpMRI was negative in all three LN positive patients.

For patient 5, mpMRI, Ga68 PSMA‐PET/CT, 3D reconstructed images of the prostate (with mpMRI and Ga68 PSMA‐PET/CT image overlap), use of the 3D images during RARP procedure, appearance of the LNs following ICG application to the prostate and postoperative pathology mapping of the prostate are shown in Figure 1.

## 4 DISCUSSION

In our study, we were able to successfully reconstruct 3D VR models by using mpMRI and Ga68 PSMA‐PET/CT images and transfer them to the surgical robot.

Initially 3D printed virtual prostate models were designed,^10^ and thereafter, 2D images (mostly mpMRI and CT) were used to reconstruct 3D VR images. Porpiglia et al reported that these 3D mpMRI reconstructed virtual models were successfully used for performing a cognitive RARP that helped to decrease (+) SM rates.^11^ Likewise, Hyde and colleagues reported that in 19% of their cases of partial nephrectomy, surgeons revised their surgical strategy following evaluating 3D virtual models.^12^ 3D mpMRI reconstructed images can be transferred either to VR headsets or to Da Vinci surgical robotic console via TilePro (please see video).

Currently, mpMRI is probably the most important imaging modality in showing cancerous foci in the prostate.^13^ In addition, Ga68 PSMA‐PET/CT is increasingly used in PCa imaging.^14^ Therefore, currently these two imaging modalities seem to be the most important ones in PCa work‐up. To the best of our knowledge, our study is the first that used 3D VR reconstructed images of the prostate combining mpMRI and Ga68 PSMA‐PET/CT images.

Growing evidence of the importance of performing radical prostatectomy (RP) in high‐risk PCa draws particular attention on this subject. Very recently, in a systematic review and meta‐analysis, RP was shown to have significantly more survival benefits than radiotherapy on cancer‐specific survival (*P* = .003) and overall survival (*P* = .002) in patients with high‐risk PCa disease.^15^ This might mean that we need to train more surgeons on robotic surgery who could be able to successfully perform RARP procedures with negative SMs in this particular group of patients.

In our series, for the patients with pT3 disease, pathological evaluation confirmed extensive tumor beyond the capsule. SM was negative in two patients with pT3aN0/N1 diseases (n = 4). For the two patients with positive SMs (patients 3 and 5), one had only a 2 mm positive SM length whereas the other one had 5 mm and 1 cm sized positive SM lengths. In patient number 3, pathology report mentioned the presence of cribriform morphology with the tumor. MRI suggested EPE, left SVI, extension to the left NVB and bilateral external iliac and obturator pelvic LNs with a size of <1 cm. Ga68 PSMA PET/CT was carried out at another center and reviewed by our nuclear medicine physician. Multiple millimeter sized Ga68 uptakes were identified on left iliac LN area at the level of the left ureter crossing iliac artery that might suggest metastasis. Pathology report mentioned that size of the metastatic LN was 6 cm and size of the metastatic focus was 2 cm in the LN. For this patient, MR more accurately showed massive extracapsular invasion on the left side of the prostate compared to Ga68 PSMA‐PET/CT, however, could not show LN involvement accurately. Significance of ICG fluorescence guided BPLND is not currently clear.^7^, ^8^ We used this approach in two patients who had pT3aN1 diseases (patients 3 and 5). This approach was successful to identify a metastatic LN during surgery in one patient.

Use of VR images as a guide and tumor navigated surgery might be helpful to achieve (−) SMs. However, microscopic evidence confirms presence (−) SMs. VR navigated RARP might increase the probability of achieving (−) SMs that might decrease excision of the preserved NVB due to (+) SM in a young patient with presumed cT3 disease who wish to preserve his nerves during a RARP procedure. Prospective studies assessing the outcomes of this approach are needed in order to make a clear conclusion that warrants further research.

Patient numbers 1, 3, and 5 did not have Neurosafe approach in our series who all had pT3 disease. SM was (−) in patient 1 who has an anterior tumor and surgery was straightforward thus tissue for frozen section was not sent. Regarding patient 5, only a 2 mm (+) SM was reported on final pathology and the surgeon did not expect to have a (+) SM for this patient. Non‐NVB sparing RARP procedure on the tumor side was straightforward and no impression of having a possible (+) SM was felt during the surgery thus tissue for frozen section was not sent. For these two patients, VR images were found to be helpful to make the decision of performing non‐NVB sparing approach on the tumor side. However, for case number 3, it was known particularly by looking at the VR and mpMRI images that there was a massive ECE on the left side and tumor was reaching almost to the levator muscle. Although a wide extrafascial excision was performed on the tumor side, (+) SM occurrence was expected before the surgery, therefore, no additional frozen was sent. Patient 2 also had pT3 disease and SMs was (−) for this patient. Overall, we had four patients with pT3 disease in the present series, one had massive ECE (patient 3). Considering that Neurosafe and/or intraoperative frozen evaluation is not performed in every institution, we think that VR tumor navigated RARP approach might be helpful particularly in T3 diseases in order to achieve (−) SMs. Combining this method with Neurosafe might further increase success.

Other previously published studies on VR models were useful to identify NVBs, capsular involvement and accessory pudendal arteries to achieve negative SMs.^4^, ^10^, ^16^, ^17^, ^18^, ^19^, ^20^, ^21^ Researchers from National Cancer Institute (NCI), USA applied a mpMRI‐based VR tool at RARP and suggested as useful in deciding to perform a wider excision to reduce (+) SMs in locally advanced PCa.^22^ We previously demonstrated that coexistence of T2 WI signs provide higher diagnostic value in predicting the grade of EPE in locally advanced PCa.^23^ A European Association of Urology (EAU) Robotic Urology Section (ERUS) survey showed that most of the participants believed that there could be a role for AR navigated tool particularly for training in robotic surgery.^24^

The limitations of our study include being a preliminary study with a small sample size without a control group. Only visible lesions on mpMRI were included. Manual data segmentation was done. An experienced radiologist, nuclear medicine physician, urologist, and engineer were required. The 3D VR reconstructed images need to be controlled by an assistant surgeon or by the console surgeon during the RARP procedure in order to present the area of interest.

In conclusion, 3D reconstructed VR models by using mpMRI and Ga68 PSMA‐PET/CT images can be accurately prepared and effectively applied during RARP that might be a useful tool for tumor navigation. Images show prostate tumors and anatomy and might be a guide for the console surgeon. They might be particularly useful in patients with locally advanced PCa that needs further study and validation.

## Supporting information

Supplementary Material
